# Supplementary material for: Evaluation of antibody serology to determine current helminth and Plasmodium falciparum infections in a co-endemic area in Southern Mozambique
Source: PLoS Negl Trop Dis. 2022 Jun 21;16(6):e0010138. doi: 10.1371/journal.pntd.0010138 (PMC9212154; doi:10.1371/journal.pntd.0010138)
Supplement: S1 Table — M: microscopy; P: qPCR; MP: microscopy and/or qPCR. Asc: A. lumbricoides; Hoo: hookworm; Tri: T. trichiura; Str: S. stercoralis; Sch: Schistosoma spp.; Sh: S. haematobium; Sm: S. mansoni; Hel: Any helminth; Pf: P. falciparum. (DOCX) [file pntd.0010138.s001.docx]

**S1 Table**. Number and percentage of positive infections by each diagnostic method and age in the study population. M: microscopy; P: qPCR; MP: microscopy and/or qPCR. Asc: *A. lumbricoides*; Hoo: hookworm; Tri: *T. trichiura*; Str: *S. stercoralis*; Sch: *Schistosoma* spp.; Sh: *S. haematobium*; Sm: *S. mansoni*; Hel: Any helminth; Pf: *P. falciparum*.

| **Method** | **Age** | **Asc** | **Hoo** | **Tri** | **Str** | **Sch** | **Sh** | **Sm** | **Hel** | **Pf** |
| --- | --- | --- | --- | --- | --- | --- | --- | --- | --- | --- |
| M | Child | 18 (4.96%) | 46 (12.67%) | 32 (8.82%) | 3 (0.83%) | 7 (1.93%) | 5 (1.38%) | 2 (0.55%) | 83 (26.6%) | - |
|  | Adult | 15 (4.26%) | 72 (20.45%) | 23 (6.53%) | 5 (1.42%) | 25 (7.1%) | 11 (3.12%) | 16 (4.55%) | 116 (38.54%) | - |
|  | All | 33 (4.62%) | 118 (16.5%) | 55 (7.69%) | 8 (1.12%) | 32 (4.48%) | 16 (2.24%) | 18 (2.52%) | 199 (30.02%) | - |
| P | Child | 24 (6.78%) | 68 (19.21%) | 64 (18.08%) | 13 (3.67%) | 14 (3.95%) | - | - | 149 (49.17%) | 32 (8.82%) |
|  | Adult | 23 (6.85%) | 116 (34.52%) | 30 (8.93%) | 58 (17.26%) | 57 (16.96%) | - | - | 205 (71.93%) | 39 (11.08%) |
|  | All | 47 (6.81%) | 184 (26.67%) | 94 (13.62%) | 71 (10.29%) | 71 (10.29%) | - | - | 354 (55.49%) | 71 (9.93%) |
| MP | Child | 28 (7.71%) | 78 (21.49%) | 70 (19.28%) | 15 (4.13%) | 19 (5.23%) | - | - | 163 (52.24%) | - |
|  | Adult | 25 (7.1%) | 123 (34.94%) | 33 (9.38%) | 59 (16.76%) | 67 (19.03%) | - | - | 220 (73.09%) | - |
|  | All | 53 (7.41%) | 201 (28.11%) | 103 (14.41%) | 74 (10.35%) | 86 (12.03%) | - | - | 383 (57.77%) | - |
